# Association between the *Bsm*I Polymorphism in the Vitamin D Receptor Gene and Breast Cancer Risk: Results from a Pakistani Case-Control Study

**DOI:** 10.1371/journal.pone.0141562

**Published:** 2015-10-30

**Authors:** Muhammad Usman Rashid, Merium Muzaffar, Faiz Ali Khan, Maria Kabisch, Noor Muhammad, Sabeen Faiz, Asif Loya, Ute Hamann

**Affiliations:** 1 Department of Basic Sciences Research, Shaukat Khanum Memorial Cancer Hospital and Research Centre (SKMCH & RC), Lahore, Pakistan; 2 Department of Human Genetics and Molecular Biology, University of Health Sciences, Lahore, Pakistan; 3 Molecular Genetics of Breast Cancer, German Cancer Research Center (DKFZ), Heidelberg, Germany; 4 Department of Physiology, University of Health Sciences, Lahore, Pakistan; 5 Department of Pathology, Shaukat Khanum Memorial Cancer Hospital and Research Centre (SKMCH & RC), Lahore, Pakistan; Ohio State University Medical Center, UNITED STATES

## Abstract

**Background:**

Vitamin D is postulated to decrease the risk of breast cancer by inhibiting cell proliferation via the vitamin D receptor (VDR). Two common single nucleotide polymorphisms (SNPs) in the *VDR* gene, rs1544410 (*Bsm*I) and rs2228570 (*Fok*I), are inconsistently associated with breast cancer risk in Caucasian populations, while data for Asians are scarce. Here, we investigated the possible contribution of these SNPs to breast cancer risk in Pakistani breast cancer patients and in controls participating in a hospital-based breast cancer case-control study (PAK-BCCC).

**Methods:**

Genotyping of the *Bsm*I and *Fok*I SNPs was performed by PCR-based restriction fragment length polymorphism (RFLP) analysis of 463 genetically enriched female breast cancer cases with known *BRCA1/2* status and in 1,012 controls from Pakistan. The association between SNP genotypes and breast cancer risk was investigated by logistic regression adjusted for potential breast cancer risk factors and stratified by *BRCA1/2* status and family history. Odds ratios (ORs) and 95% confidence intervals (CIs) were reported.

**Results:**

The b allele of the *Bsm*I was associated with an increased breast cancer risk (per b allele OR 1.28, 95% CI 1.09–1.49, *P* = 0.003). Subgroup analysis revealed that this effect was restricted to *BRCA1/2* non-carriers (per b allele OR 1.33, 95% CI 1.11–1.59, *P* = 0.002) and was stronger in those who reported a positive family history of breast and/or ovarian cancer (per b allele OR 1.64, 95% CI 1.20–2.22, *P* = 0.002). No association with breast cancer risk was detected for the *Fok*I SNP.

**Conclusions:**

The *Bsm*I polymorphism in the *VDR* gene may be associated with an increased breast cancer risk in Pakistani women negative for *BRCA1/2* germline mutations.

## Introduction

Breast cancer is the major cause of morbidity and mortality in women worldwide. In Pakistan, this disease constitutes a main public health issue as it accounts for 40% of all female cancers (Globocan 2012; http://globocan.iarc.fr/). Variants in the high and moderate penetrance breast cancer susceptibility genes *BRCA1*, *BRCA2*, *TP53*, *CHEK2* and *RAD51C* account for approximately 20% of hereditary breast cancer in Pakistan [1–4]. Data on the contribution of low-penetrance variants to the disease are lacking at present.

In recent years, a role for vitamin D in the development of breast cancer has been increasingly recognized. Evidence from several epidemiological studies confirms that low serum 25-hydroxyvitamin D3 levels—representing an integrative measure for vitamin D from diet, dietary supplements, and skin production—are associated with an increased breast cancer risk [5–7]. The effects of the active form of vitamin D, 1α, 25-dihydroxyvitamin D3, are mediated through the vitamin D receptor (VDR). This receptor is a ligand-dependent transcription factor that belongs to the family of nuclear receptors [8].

Activation of VDR by 1α, 25-dihydroxyvitamin D3 results in heterodimerization with the retinoid X receptor and binding to cognate vitamin D response elements in target genes involved in cellular differentiation, cell growth, apoptosis, inflammation, and immune modulation [9–11]. Vitamin D-liganded VDR displays anti-proliferative activities in many tumor types, as do activated members of the p53 family, through the induction of cell cycle arrest, senescence, differentiation, and apoptosis [12]. Increasing evidence supports crosstalk between the vitamin D and p53 signaling pathways. All three members of the p53 family—p53, p63, and p73—have been shown to transactivate the *VDR* gene [13,14], while *VDR*, in turn, regulates several genes that are also targeted by the proteins of the p53 family, including *p21*, *Bax*, *Bcl-2*, and *MDM2* [12,15,16].

Breast tumors show decreased levels of VDR expression [17], which may be due to genetic variants in the *VDR* gene. Alterations in VDR expression and activity may alter the ability of the active form of vitamin D to induce the transcription of VDR target genes. This may happen even in women who are not vitamin D deficient, and may lead to a deregulation of the uptake, metabolism, and serum level of vitamin D.

Several *VDR* variants have been identified that may influence breast cancer risk. The most frequently studied SNPs are the restriction fragment length polymorphisms (RFLPs) rs1544410 [18] and rs2228570 [19], which are defined by the restriction endonucleases *Bsm*I and *Fok*I, respectively. The *Bsm*I SNP is located in intron 8 near the 3´end of the *VDR* gene. Whether it influences the expression or activity of the VDR protein is unclear, but it is in strong linkage disequilibrium with a polyadenosine (poly(A)) microsatellite repeat in the 3´untranslated region in Caucasians, Chinese, and Japanese-Americans [20], which may affect VDR translational activity or mRNA stability. The *Fok*I site is located in the 5´promoter region and changes the first of the two possible translation initiation sites, resulting in VDR proteins of different lengths. The f allele is three amino acid residues longer than the F allele and is transcriptionally less active [21].

Many studies performed on Caucasian populations regarding the association of the *Bsm*I and *Fok*I SNPs with breast cancer risk have yielded inconsistent results [22–25]. Few studies have been conducted on Asian populations. The *Fok*I SNP showed no associations in a large Chinese study [26] or in a small study conducted in Iran [27], while an association was found in Japanese-American women from the Hawaii-Los Angeles Multiethnic Cohort (MEC) study [28]. The *Bsm*I SNP showed an association in the Iranian study [27] and in Japanese-American women from the MEC study [28], but not in two Chinese studies [26,29].

Given the scant and inconsistent data from Asian populations, we investigated whether the *Bsm*I and *Fok*I polymorphisms are associated with breast cancer risk in an Asian population from Pakistan. We genotyped 463 genetically enriched breast cancer cases and 1,012 female controls participating in the hospital-based Pakistani breast cancer case-control study (PAK-BCCC).

## Materials and Methods

### Ethics statement

The study was approved by the institutional review board of the Shaukat Khanum Memorial Cancer Hospital and Research Centre (SKMCH & RC) (Approval # 00-16-05-06). All participants signed informed written consent prior to providing a blood sample.

### Study participants

The PAK-BCCC study is an ongoing study being conducted at the SKMCH & RC in Lahore, Pakistan. The SKMCH & RC is the leading referral and cancer treatment hospital in Lahore. It provides financially-supported treatment for more than 75% of its patients, who come from all over the country. Since the hospital is situated in the provincial capital of Punjab, a province that accounts for approximately 60% of the total Pakistani population, 74.6% of its patients are of Punjabi ethnicity (SKMCH & RC Collective Cancer Registry Report from December 1994 to December 2013; http://www.shaukatkhanum.org.pk/research/cancer-registry-and-clinical-data-management.html). From June 2001 to October 2011, 463 women with “genetically enriched” breast cancer and 1,012 controls were recruited.

“Genetically enriched” breast cancer cases were defined as those: (1) aged younger or equal 30 years at diagnosis, (2) with one first-degree or second-degree (through a male) female relative affected with breast cancer (at least one diagnosed ≤ 50 years of age), (3) with at least two female relatives affected with breast cancer (at least one diagnosed ≤ 50 years of age), or (4) with at least one ovarian cancer at any age [1]. The numbers of index cases by family history are shown in S1 Table. All cases were comprehensively screened for germline mutations in the *BRCA1* and *BRCA2* genes, as previously described [1]. Of these study subjects, deleterious *BRCA1/2* mutations were identified in 110 cases (M.U. Rashid, unpublished data). Of these 463 breast cancer cases, 138—including 24 with *BRCA1*/2 mutations—have been described in the initial publication [1]. Information on histopathological tumor characteristics, including histology (ductal, lobular, mixed ductal and lobular), tumor size, lymph node status, histological grade, estrogen receptor (ER), progesterone receptor (PR), and human epidermal growth factor receptor 2 (HER2/neu) status, was collected from medical records and pathology reports.

Female controls were enrolled simultaneously with cases when they fulfilled the following criteria: (1) age 18 years or older, (2) cancer-free, i.e. no personal history of any malignancy, and (3) no family history of breast and ovarian cancer. Controls were either attendants of patients under treatment at the hospital or visiting for non-cancer medical reasons or comprehensive medical checkup. The health status of controls was documented by collecting information about non-cancer diseases. The majority of the controls were spouses, friends, colleagues, neighbors, or relatives of hospital-registered patients. They were contacted by a well-trained study coordinator at the waiting areas of outpatient clinics, chemotherapy bays, or inpatient departments, who informed them about the study and invited them to participate. None of the 1,012 contacted controls refused participation.

All study participants filled out a specifically designed risk factors questionnaire that included age at diagnosis (cases)/interview (controls), age at menarche, number of full-term pregnancies (FTPs), age at first FTP, history of breast feeding, duration of breast feeding, menopausal status, use of oral contraceptives (OC) or menopausal hormonal therapy (HT), body mass index (BMI), smoking, ethnicity, and family history of at least one first degree relative with breast and/or ovarian cancer.

### Extraction of genomic DNA and genotyping

Genomic DNA was extracted from nine ml of whole blood using the Puregene™ (Gentra Systems, Minneapolis, USA) kit according to the manufacturer’s instructions. The extracted DNA was rehydrated in DNA hydration solution (10 mM Tris, 1 mM EDTA, pH 7–8; Gentra Systems, Minneapolis, USA) and stored at -70°C until further use.

Genotyping of rs1544410 (*Bsm*I G/b>A/B) and rs2228570 (*Fok*I T/f>C/F) in *VDR* by PCR-based RFLP analysis was performed mainly according to the protocols described previously [30,31]. *Bsm*I and *Fok*I alleles were defined by capital letters (B = A and F = C, respectively) in the absence of the restriction site and by small letters (b = G and f = T, respectively) where the restriction site was present. PCR reactions were carried out in a 20 μl volume containing 25 ng of genomic DNA, 1x PCR buffer (Qiagen, Hilden, Germany), 3.0 mM MgCl_2_, 0.1 μM of each primer, 250 μM of each dNTP (Invitrogen, Carlsbad CA, USA) and 1 unit HotStarTaq DNA polymerase (Qiagen, Hilden, Germany). After an initial 15 minutes at 95°C, DNA was amplified by 35 cycles of 1 minute at 94°C, 1 minute at 66°C (for *Bsm*I) or 60°C (for *Fok*I), 1 minute at 72°C, and a final extension step of 10 minutes at 72°C. Amplified fragments were digested with 2.5 units *Bsm*I (Fermentas, Vilnius, Lithuania) and 1 unit *Fok*I (Fermentas, Vilnius, Lithuania) in a total volume of 15 μl for 3 hours at 37°C and 55°C, respectively. Digested DNA fragments were separated by size on a 2% agarose gel (Sigma-Aldrich, Steinheim, Germany) containing ethidium-bromide (Invitrogen, Carlsbad CA, USA) and were visualized under short-wave UV light using a Molecular Imager^®^ Gel Doc^™^ XR System (Bio-Rad Laboratories, Hercules CA, USA). DNA from individuals with the BB genotype of the *Bsm*I appeared on the gel as single 823 bp fragment, from bb homozygotes as two fragments of 646 and 177 bp and from Bb heterozygotes as three fragments of 823, 646, and 177 bp. For the *Fok*I, the corresponding fragments were 273 bp for FF homozygotes, 197 and 76 bp for ff homozygotes and 273, 197, and 76 bp for Ff heterozygotes (S1A and S2A Figs). Each experiment included multiple controls, including a water blank.

Genotypes were scored by two independent persons, who were blinded to the case-control status. Overall call rates for the *Bsm*I and *Fok*I SNPs were 97.0% and 96.8%, respectively. Concordance rates of 453/1,475 (31%) duplicate samples for *Bsm*I and 157/1,475 (11%) samples for *Fok*I were 100% each. Genotyping accuracy was assessed by sequencing of several samples with detected polymorphic variants using an automated 3500 Genetic Analyzer (Applied Biosystems, California, USA) according to the manufacturer’s instructions (S1B–S1D and S2B–S2D Figs).

### Statistical analysis

The two genotyped *VDR* SNPs were tested for Hardy-Weinberg equilibrium (HWE) using Pearson’s Chi-square test. Associations between each SNP and overall breast cancer risk (all cases *vs*. all controls) were analyzed by logistic regression adjusted for the following twelve breast cancer risk factors: age (age at diagnosis for cases, age at recruitment for controls), age at menarche, number of FTPs, age at first FTP, history of breast feeding, duration of breast feeding, menopausal status, use of OC, use of menopausal HT, BMI, smoking, and ethnicity. Additionally, subgroup analyses were carried out by stratifying cases according to *BRCA1/2* mutation status, family history, and menopausal status. The subgroup of cases that did not carry a *BRCA1/2* mutation and the subgroup of cases that did carry a *BRCA1/2* mutation were separately compared to the control group. The subgroups of affected *BRCA1/2* non-carriers with and without a family history of breast and/or ovarian cancer in first-degree relatives were also separately compared to the control group. Further, pre- and post-menopausal women, irrespective of their *BRCA1/2* mutation status, were compared to any control. Associations between each SNP and seven histopathological tumor characteristics—morphology, tumor size, nodal status, histological grade, ER, PR, and HER2/neu status—were investigated in cases only, using either binary, multinomial, or ordinal logistic regression and including the twelve breast cancer risk factors mentioned above as covariates. To investigate sensitivity of obtained association results all breast cancer risk analyses were additionally carried out for a) a reduced set of covariates including only those variables with an observed difference between cases and controls (*P*<0.05), and b) the subgroup of controls having no documented disease (cases *vs*. healthy controls).

In all association analyses, we considered an additive penetrance model for *Bsm*I and *Fok*I and defined the homozygous genotype of the more prevalent allele (BB and FF, respectively) as reference genotype. These genotypes were also used as reference group in various other studies [27,31–34]. Per allele odds ratios (ORs) and corresponding 95% confidence intervals (CIs) were reported. Multiple testing was taken into account by adjusting the significance levels *a posteriori* using Bonferroni correction. In case of statistically significant findings for the overall breast cancer risk analysis, the penetrance models 3-genotype, recessive and dominant, were also investigated. All penetrance models were then compared based on Akaike’s information criterion (AIC). Statistical analyses were performed using SAS software, version 9.2 (SAS Institute Inc., Cary, NC, USA).

## Results

### Characteristics of the study participants

The present study included 463 genetically enriched breast cancer cases and 1,012 controls from Pakistan, recruited at the SKMCH & RC. Breast cancer cases were diagnosed at a median age of 30 years (range 19–73 years). The median age of controls at study entry was 34 years (range 18–78 years). Of the controls, 74.3% were healthy, 5.9% suffered from hypertension, 4.4% from allergy, 2.2% from diabetes mellitus, 2.2% from asthma, and 1.9% from both hypertension and diabetes mellitus. The remaining 7.9% of controls suffered from uncommon diseases, which occurred in 1% or less of the controls. The health status was unknown for 1.2%.

Comparison of the distribution of selected clinical and epidemiological parameters between cases and controls revealed significant differences in age, number of FTPs, and duration of breast feeding (two-sided Chi-square test, *P*<0.0001). A difference was also found in age at menarche (*P* = 0.04), age at first FTP (*P* = 0.03), menopausal status (*P* = 0.02), and ethnicity (*P* = 0.04). The characteristics of the PAK-BCCC study participants are shown in Table 1.

**Table 1:** Characteristics of the PAK-BCCC study participants.

Characteristic	Cases (N = 463)	Controls (N = 1,012)	*P*-value^1^
	n (%)	n (%)	
Age (years)^2^			**<0.0001**
<20	4 (0.9)	18 (1.8)	
20–29	211 (45.6)	342 (33.8)	
30–39	133 (28.7)	282 (27.9)	
40–49	72 (15.6)	222 (21.9)	
50–59	32 (6.9)	111 (11.0)	
≥60	11 (2.4)	30 (3.0)	
Unknown	0 (0)	7 (0.7)	
Age at menarche (years)			**0.04**
<12	18 (3.9)	57 (5.6)	
12	92 (19.9)	181 (17.9)	
13	162 (35.0)	300 (29.6)	
≥14	176 (38.0)	451 (44.6)	
Unknown	15 (3.2)	23 (2.3)	
No. of FTPs			**<0.0001**
0	129 (27.9)	323 (31.9)	
1	56 (12.1)	56 (5.5)	
2	76 (16.4)	105 (10.4)	
3	93 (20.1)	159 (15.7)	
≥4	105 (22.7)	361 (35.7)	
Unknown	4 (0.9)	8 (0.8)	
Age at first FTP (years)			**0.03**
Nulliparous	129 (27.9)	323 (31.9)	
<20	63 (13.6)	187 (18.5)	
20–24	144 (31.1)	251 (24.8)	
25–29	101 (21.8)	190 (18.8)	
≥30	20 (4.3)	47 (4.6)	
Unknown	6 (1.3)	14 (1.4)	
History of breast feeding			0.52
Ever	311 (67.2)	649 (64.1)	
Never	147 (31.8)	352 (34.8)	
Unknown	5 (1.1)	11 (1.1)	
Duration of breast feeding (months)			**<0.0001**
Never	147 (31.8)	352 (34.8)	
<12	46 (9.9)	58 (5.7)	
12–23	49 (10.6)	67 (6.6)	
≥24	208 (44.9)	524 (51.8)	
Unknown	13 (2.8)	11 (1.1)	
Menopausal status			**0.02**
Pre-menopausal	349 (75.4)	825 (81.5)	
Post-menopausal	107 (23.1)	178 (17.6)	
Unknown	7 (1.6)	9 (0.9)	
OC use			0.22
Ever	40 (8.6)	118 (11.7)	
Never	419 (90.5)	885 (87.5)	
Unknown	4 (0.9)	9 (0.9)	
HT use			0.84
Ever	3 (0.7)	9 (0.9)	
Never	455 (98.3)	994 (98.2)	
Unknown	5 (1.1)	9 (0.9)	
BMI (kg/m^2^)			0.17
Underweight (<18.5)	21 (4.5)	69 (6.8)	
Normal (18.5–24.9)	213 (46.0)	446 (44.1)	
Overweight (25–29.9)	126 (29.2)	299 (29.5)	
Obese (≥30)	82 (17.7)	169 (16.7)	
Unknown	21 (4.5)	29 (2.9)	
Smoking			0.39
Ever	8 (1.7)	12 (1.2)	
Never	448 (96.8)	991 (97.9)	
Unknown	7 (1.5)	9 (0.9)	
Ethnicity			**0.04**
Punjabi	337 (72.8)	781 (77.2)	
Pathan	60 (13.0)	93 (9.2)	
Urdu speaking	21 (4.5)	46 (4.5)	
Kashmiri	17 (3.7)	18 (1.8)	
Other	23 (5.0)	67 (6.5)	
Unknown	5 (1.1)	7 (0.7)	

FTP: full term pregnancy, OC: oral contraceptives, HT: hormone therapy, BMI: body mass index.

^1^Probability value based on a two-sided Chi-square test. *P*-values below 0.05 are marked in bold.

^2^Age at diagnosis for cases or age at recruitment for controls.

### Association of the *VDR* SNPs with breast cancer risk

We genotyped the *Bsm*I and *Fok*I SNPs in the PAK-BCCC study population. Among the controls the frequency of the minor f allele of the *Fok*I and the b allele of the *Bsm*I was 0.21 and 0.48, respectively. The frequency of the f allele of the *Fok*I was similar to that reported in the Punjabi population of the 1000 Genomes Project, while the frequency of the b allele of the *Bsm*I was lower (0.20 and 0.56, respectively) [35]. The distribution of genotypes in the control group was consistent with HWE for the *Fok*I SNP (*P* = 0.466), but deviated from HWE for the *Bsm*I SNP in the whole control group, in the subgroup of only healthy controls, and in the subgroup of controls of only Punjabi ethnicity (*P*<0.0001) (Table 2). Our study had 80% power to identify ORs of 1.45 and 1.37, assuming a minor allele frequency (MAF) of 0.21 for *Fok*I and 0.48 for *Bsm*I, respectively (two-sided Chi-square test, α = 0.05).

**Table 2:** Genotype frequencies of the *Fok*I and *Bsm*I SNPs.

SNP	Cases (N = 463)	All controls (N = 1,012)	Healthy controls (N = 752)	Punjabi controls (N = 781)
	n (%)	n (%)	n (%)	n (%)
*Fok*I (*F>f*)				
*FF*	284 (61.3)	630 (62.3)	466 (62.0)	496 (63.5)
*Ff*	159 (34.3)	332 (32.8)	252 (33.5)	250 (32.0)
*ff*	20 (4.3)	50 (4.9)	34 (4.5)	35 (4.5)
HWE *P*-value^1^	0.703	0.466	0.993	0.626
*Bsm*I (*B>b*)				
*BB*	118 (25.5)	320 (31.6)	233 (31.0)	261 (33.4)
*Bb*	189 (40.8)	408 (40.3)	310 (41.2)	304 (38.9)
*bb*	156 (33.7)	284 (28.1)	209 (27.8)	216 (27.7)
HWE *P*-value^1^	**0.0001**	**<0.0001**	**<0.0001**	**<0.0001**

^1^Probability value based on Pearson’s Chi-square test.

*P*-values below 0.05 are marked in bold.

The b allele of the *Bsm*I was associated with an increased risk of breast cancer (per b allele OR 1.28, 95% CI 1.09–1.49, *P* = 0.003) (Table 3). The additive penetrance model was the best representation for this association (additive model AIC = 1713, 3-genotype model AIC = 1715, recessive model AIC = 1716, dominant model AIC = 1716). Stratified analyses by *BRCA1/2* status revealed a marginally stronger association in cases that did not carry a *BRCA1/2* mutation (per b allele OR 1.33, 95% CI 1.11–1.59, *P* = 0.002), but no association in *BRCA1/2* mutation carriers (Table 3). Further analyses stratified by family history and menopausal status showed that the association of the *Bsm*I SNP with breast cancer risk in *BRCA1/2* non-carriers was maintained in patients with no family history of breast and/or ovarian cancer (per b allele OR 1.27, 95% CI 1.02–1.56, *P* = 0.03) and in those who were premenopausal (per b allele OR 1.25, 95% CI 1.03–1.54, *P* = 0.03) and was further increased in patients with a positive family history of breast and/or ovarian cancer (per b allele OR 1.64, 95% CI 1.20–2.22, *P* = 0.002) and in those who were postmenopausal (per b allele OR 1.59, 95% CI 0.98–2.56, *P* = 0.06) (Table 4). No association of the *Fok*I SNP was noted in overall breast cancer risk analysis or in the stratified analyses (Tables 3 and 4). Sensitivity analyses including a reduced covariate model (Tables 3 and 4) and only healthy individuals as controls (S2 and S3 Tables) revealed similar effects of the *Fok*I and *Bsm*I SNPs, which confirmed breast cancer association results.

**Table 3:** Estimated ORs for the association of *Fok*I and *Bsm*I SNPs with breast cancer by *BRCA1/2* status (complete and reduced covariate model).

	Controls	Cases								
	(N = 1,012)	Any *BRCA1/2* status (N = 463)			*BRCA1/2* non-carriers (N = 353)			*BRCA1/2* carriers (N = 110)		
Genotype/Allele	n (%)	n (%)	OR (95% CI)^1^ ^,^ ^2^	*P-*value^3^	n (%)	OR (95% CI)^1^ ^,^ ^2^	*P-*value^3^	n (%)	OR (95% CI)^1^ ^,^ ^2^	*P-*value^3^
*Fok*I (*F>f*)										
*FF*	630 (62.3)	284 (61.3)	0.99 (0.80–1.23)^1^	0.94	218 (61.8)	0.97 (0.77–1.23)^1^	0.82	66 (60.0)	1.02 (0.71–1.48)^1,2^	0.90
*Ff*	332 (32.8)	159 (34.3)	0.98 (0.80–1.21)^2^	0.88	119 (33.7)	0.97 (0.77–1.22)^2^	0.80	40 (36.4)	1.03 (0.72–1.48)^2^	0.87
*ff*	50 (4.9)	20 (4.3)			16 (4.5)			4 (3.6)		
*Bsm*I (*B>b*)										
*BB*	320 (31.6)	118 (25.5)	1.28 (1.09–1.49)^1^	**0.003**	89 (25.2)	1.33 (1.11–1.59)^1^	**0.002**	29 (26.4)	1.18 (0.89–1.54)^1^	0.26
*Bb*	408 (40.3)	189 (40.8)	1.25 (1.08–1.47)^2^	**0.004**	141 (39.9)	1.29 (1.09–1.54)^2^	**0.003**	48 (43.6)	1.16 (0.88–1.54)^2^	0.27
*bb*	284 (28.1)	156 (33.7)			123 (34.8)			33 (30.0)		

^1^Complete model: Odds ratios (ORs) with corresponding 95% confidence intervals (CIs) adjusted for age, age at menarche, number of FTPs, age at first FTP, history of breast feeding, duration of breast feeding, menopausal status, OC use, HT use, BMI, smoking and ethnicity.

^2^Reduced model: ORs with corresponding 95% CIs adjusted for age, age at menarche, number of FTPs, age at first FTP, duration of breast feeding, menopausal status and ethnicity.

^3^Probability value based on logistic regression and an additive penetrance model. *P*-values below 0.05 are marked in bold.

**Table 4:** Estimated ORs for the association of the *Fok*I and *Bsm*I SNPs with breast cancer in *BRCA1/2* non-carriers by family history and menopausal status (complete and reduced covariate model).

	Controls	Cases											
	(N = 1,012)	Without family history (N = 247)			With a family history^1^ (N = 106)			Premenopausal (N = 267)			Postmenopausal (N = 80)		
Genotype	n (%)	n (%)	OR (95% CI)^2^ ^,^ ^3^	*P-*value^4^	n (%)	OR (95% CI)^2^ ^,^ ^3^	*P-*value^4^	n (%)	OR (95% CI)^2^ ^,^ ^3^	*P-*value^4^	n (%)	OR (95% CI)^2^ ^,^ ^3^	*P-*value^4^
*Fok*I (*F>f*)													
*FF*	630 (62.3)	154 (62.4)	0.94 (0.71–1.24)^2^	0.65	64 (60.4)	1.01 (0.69–1.48)^2^	0.97	167 (62.6)	0.94 (0.72–1.24)^2^	0.67	46 (57.5)	0.96 (0.52–1.78)^2^	0.89
*Ff*	332 (32.8)	80 (32.4)	0.94 (0.71–1.22)^3^	0.63	39 (36.8)	1.01 (0.69–1.48)^3^	0.97	91 (34.1)	0.92 (0.71–1.20)^3^	0.55	28 (35.0)	1.04 (0.61–1.78)^3^	0.89
*ff*	50 (4.9)	13 (5.3)			3 (2.8)			9 (3.4)			6 (7.5)		
*Bsm*I (*B>b*)													
*BB*	320 (31.6)	66 (26.7)	1.27 (1.02–1.56)^2^	**0.03**	23 (21.7)	1.64 (1.20–2.22)^2^	**0.002**	67 (25.1)	1.25 (1.03–1.54)^2^	**0.03**	19 (23.8)	1.59 (0.98–2.56)^2^	0.06
*Bb*	408 (40.3)	100 (40.5)	1.23 (1.01–1.52)^3^	0.05	41 (38.7)	1.59 (1.18–2.13)^3^	**0.003**	105 (39.3)	1.22 (1.01–1.47)^3^	**0.04**	34 (42.5)	1.61 (1.03–2.56)^3^	**0.04**
*bb*	284 (28.1)	81 (32.8)			42 (39.6)			95 (35.6)			27 (33.7)		

^1^Family history of breast and/or ovarian cancer in first-degree relatives.

^2^Complete model: Odds ratios (ORs) with corresponding 95% confidence intervals (CIs) adjusted for age, age at menarche, number of FTPs, age at first FTP, history of breast feeding, duration of breast feeding, menopausal status, OC use, HT use, BMI, smoking and ethnicity.

^3^Reduced model: ORs with corresponding 95% CIs adjusted for age, age at menarche, no. of FTPs, age at first FTP, duration of breast feeding, menopausal status and ethnicity.

^4^Probability value based on logistic regression and an additive penetrance model. *P*-values below 0.05 are marked in bold.

Bonferroni correction for multiple testing (global significance level divided by the number of performed tests in association analyses: 0.05/14) resulted in an adjusted significance level of 0.004. Hence, the association of the *Bsm*I SNP with: i) overall breast cancer risk, ii) breast cancer risk in *BRCA1/2* non-carriers, and iii) breast cancer risk in *BRCA1/2* non-carriers with a positive family history of breast and/or ovarian cancer, reached statistical significance.

### *VDR* SNPs and histopathological characteristics of breast tumors

Table 5 shows the distribution of cases according to the available tumor characteristics. The majority of cases presented with a grade 3 breast tumor (64.4%) of ductal histology (85.1%), negative for ER (53.8%), PR (53.3%), and HER2/neu (60.7%) expression and with a positive lymph node status (51%).

**Table 5:** Histopathological parameters of the breast tumors of the cases participating in the PAK-BCCC study.

Characteristics	Cases (N = 463)
	n (%)
Histological type	
Ductal	394 (85.1)
Lobular	13 (2.8)
Ductolobular	9 (1.9)
Other	22 (4.8)
Unknown	25 (5.4)
Tumor size	
T0	5 (1.1)
T1	70 (15.1)
T2	199 (43.0)
T3	70 (15.1)
T4	7 (1.5)
Unknown	112 (24.2)
Nodal status	
N0	176 (38.0)
≥N1	236 (51.0)
Unknown	51 (11.0)
Histological grade	
G1	3 (0.6)
G2	102 (22.0)
G3	298 (64.4)
Unknown	60 (13.0)
ER status	
Negative	249 (53.8)
Positive	181 (39.1)
Unknown	33 (7.1)
PR status	
Negative	247 (53.3)
Positive	182 (39.3)
Unknown	34 (7.3)
HER2/neu status	
Negative	281 (60.7)
Positive	128 (27.6)
Unknown	54 (11.7)

ER: estrogen receptor, PR: progesterone receptor, HER2/neu: human epidermal growth factor receptor 2.

In order to analyze whether the two *VDR* SNPs affect tumor progression, *Bsm*I and *Fok*I genotypes were correlated with histopathological tumor characteristics (S4 Table). No association was observed for any of the investigated tumor parameters.

## Discussion

This is the first large study that assessed associations of low-penetrance variants with breast cancer risk in Pakistan. Associations of the *Bsm*I and *Fok*I SNPs in the *VDR* gene with breast cancer risk and histopathological tumor parameters were examined in a data set from women participating in a large hospital-based PAK-BCCC study that included 463 genetically enriched breast cancer cases and 1,012 controls. While no association with breast cancer risk was observed for the *Fok*I SNP, the b allele of the *Bsm*I was associated with a 33% increase in breast cancer risk in *BRCA1/2* non-carriers and a 64% increase in *BRCA1/2* non-carriers with a family history of breast and/or ovarian cancer. Inclusion of only healthy controls in the association analysis yielded a similar association signal implying that the inclusion of controls affected with VDR-related diseases did not confound our results and data interpretation.

To date, few studies with at least 100 cases and 100 controls have assessed associations of the *Fok*I and/or *Bsm*I SNP(s) with breast cancer risk in Asians. For the *Fok*I SNP, no associations were reported in a large study that included 2,919 cases and 2,323 controls from China [26] and another small study on 140 cases and 156 controls from Iran [27], which is in line with the results of the present study. Our results are also consistent with those obtained for other ethnicities, such as African-Americans [28,36] and Hispanics [28,33,37]. Contrasting results were obtained in another study among Japanese-American women from the MEC study, including 401 cases and 405 controls, which showed an association of the ff genotype with an increased breast cancer risk [28]. In Caucasians, the ff genotype was also associated with an increased risk of breast cancer and cancer at any site, in several single studies and comprehensive meta-analyses [22,24,25].

For the *Bsm*I SNP, no associations were found in the Chinese study conducted by Dorjgochoo and colleagues [26] and in another small Chinese study on 146 cases and 320 controls [29]. In contrast, in Japanese-American women from the MEC study, the B allele was associated with a decreased breast cancer risk [28], while in the Iranian study, the b allele was associated with an increased breast cancer risk [27]. Our study, performed on a large number of cases and controls from the Pakistani population, confirmed the positive association between the b allele of the *Bsm*I and the increased risk of developing breast cancer. This association remained statistically significant after correction for multiple testing. This effect was restricted to the subgroup of *BRCA1/2* non-carriers, indicating that the biological mechanisms underlying the association of the *Bsm*I SNP with breast cancer risk may be subverted in *BRCA1/2* mutation carriers. Individual variants are expected to show stronger effects in familial, genetically enriched cases [38]. Here, we found that the association of the b allele of the *Bsm*I with breast cancer risk was further increased in *BRCA1/2* non-carriers who reported a positive family history of breast and/or ovarian cancer.

Varying results for the associations of the *Bsm*I SNP and breast cancer risk were reported in other ethnic populations. One Hispanic study showed an association of the B allele with an increased breast cancer risk [37], whereas other recent studies reported no associations [28,33]. In Caucasians, associations of the BB genotype with a decreased breast cancer were reported [39,40], while no associations were found in the largest single study performed so far or in recent meta-analyses [23,25,28]. An association of the Bb and BB genotype with a decreased cancer risk for any site was observed in a recent comprehensive meta-analysis by Raimondi and colleagues [23]. No similar association was observed in African-Americans [28,36]. Results of these studies were inconsistent, which possibly may reflect the limited sample size of several of these studies and heterogeneity in the study designs, including the different ethnicities of the study populations.

In the control group in the present study, we observed a deviation from the expected HWE for the *Bsm*I SNP, but not the *Fok*I SNP. Deviation from HWE could be attributed to a number of factors, including genotype misclassification, chance, undetected ethnic diversity, inbreeding caused by consanguinity, and non-random-mating, selection, or migration [41]. *Bsm*I genotype misclassification is unlikely to cause departure from HWE in the present study, as genotyping was done while blinded to the case-control status, the concordance rate of 31% of the duplicate samples was 100%, and the three genotypes were confirmed in some samples using bidirectional DNA sequencing as a second genotyping method. Departure from HWE was also observed when only controls of Punjabi ethnicity, constituting the largest ethnic group, were considered. This may be due to unrecognized population stratification since the Punjabis represent an ethnically diverse population comprising the local Punjabis, immigrants from Indian Punjab who settled in Pakistan after the partition of the Indian subcontinent in 1947 and invaders, who also passed through Punjab in the past 2,000 years and mixed with previous settlers [42]. No evidence came to light that departure from HWE was due to inclusion of affected controls, since it remained in the subgroup of healthy controls. Deviation from HWE is unlikely to be due to inclusion of related study participants, since relatedness of all study participants was examined by pedigree analysis and surname comparison. For individuals with identical surnames, the names of the parents, the names and numbers of siblings and aunts/uncles, and the home addresses were additionally checked in order to identify related individuals. A reason for the observed departure from HWE in the Pakistani study may be inbreeding by consanguinity. Pakistan has one of the highest rates of consanguinity in the world [http://www.consang.net/index.php/Global_prevalence]. The overall frequency of consanguineous marriages, mainly first cousin marriages, ranges from 56% to 66.4% [43–45]. Notably, deviation from HWE for the *Bsm*I SNP in controls has also been previously observed in other association studies conducted in Caucasian and African-American populations [28,31,46].

The present study has a number of strengths. We used genetically enriched cases, which are more likely to carry at-risk alleles than are unselected breast cancer patients. We also used at least two controls per case, which improves the power of the study [38]. Moreover, knowledge of the *BRCA1/2* mutation status allowed risk estimates in homogenous patient groups of *BRCA1/2* carriers and non-carriers.

The study has certain limitations. It is a hospital-based case-control study, which may have introduced some selection bias. The potential for some survival bias also existed, since only 73% of cases were recruited within the first year of diagnosis. Furthermore, we did not investigate the potential modifying effects of environmental factors, such as dietary factors, on the observed association of the *Bsm*I SNP and breast cancer risk. We also did not measure vitamin D serum levels to examine interactions between vitamin D levels and *VDR* SNPs in relation to breast cancer risk. A high prevalence of vitamin D deficiency has been reported in Pakistani women [47], newly diagnosed Pakistani breast cancer patients [48], and Pakistani immigrants living in Norway [49] and the UK [50]. Vitamin D deficiency is associated with several diseases, including hypertension, allergy, and diabetes mellitus [51–53]. In the present study, 16% of the controls suffered from vitamin D deficiency-related diseases. However, this is unlikely to have influenced our results, as genotype frequencies in the whole control group did not differ from those of the subgroup of healthy controls for both *VDR* SNPs. Another limitation of the study is that we did not investigate candidate gene variants in other genes of the vitamin D pathway, such as those encoding vitamin D binding protein (DBP) or enzymes involved in vitamin D activation and degradation (CYP2R1, CYP27B1, CYP27A1, and CYP24A1) [54,55], which could also be associated with breast cancer risk.

In conclusion, our study provides evidence that the b allele of the *Bsm*I in the *VDR* gene may be associated with an increased breast cancer risk in Pakistani women who are negative for *BRCA1/2* germline mutations. Future large studies in Asia are warranted to validate our findings.

## Supporting Information

S1 Fig Genotyping of the *VDR Bsm*I (rs1544410, G/b>A/B) polymorphism by PCR-based RFLP and direct DNA sequencing analysis. (**A**) PCR-RFLP products were separated on a 2% agarose gel containing ethidium bromide and scored by UV visualization. Lane 1: DNA marker (100 bp); lanes 2, 4, 7, 9, 11: AA genotype; lanes 3, 5, 8, 10: AG genotype; lanes 6, 12, 13: GG genotype (**B-D**) Sequencing profiles of control DNA samples showing the sequence of the reverse strand of part of intron 8 near the 3’end of the *VDR* gene with the C to T nucleotide change. (**B**) CC genotype; (**C**) CT genotype; (**D**) TT genotype.
(TIF)

S2 Fig Genotyping of the *VDR Fok*I (rs2228570, T/f>C/F) polymorphism by PCR-based RFLP and direct DNA sequencing analysis. (**A**) PCR-RFLP products were separated on a 2% agarose gel containing ethidium bromide and scored by UV visualization. Lane 1: DNA marker (50 bp); lanes 2, 7, 9, 10, 12, 13: CC genotype; lanes 3, 5, 6, 8, 11: CT genotype; lane 4: TT genotype (**B-D**) Sequencing profiles of control DNA samples showing the sequence of the reverse strand of part of exon 2 of the *VDR* gene with the A to G nucleotide change. (**B**) AA genotype; (**C**) AG genotype; (**D**) GG genotype.
(TIF)

S1 Table Family history of index cases genotyped for the *VDR Fok*I and *Bsm*I SNPs. (DOCX)

S2 Table Estimated ORs for the association of *Fok*I and *Bsm*I SNPs with breast cancer by *BRCA1/2* status (all cases *vs*. healthy controls, complete covariate model). (DOCX)

S3 Table Estimated ORs for the association of the *Fok*I and *Bsm*I SNPs with breast cancer in *BRCA1/2* non-carriers by family history and menopausal status (all cases *vs*. healthy controls, complete covariate model). (DOCX)

S4 Table Estimated ORs for the association of *Fok*I and *Bsm*I SNPs with breast cancer by histopathological tumor characteristics. (DOCX)

## Abbreviations

AIC: Akaike’s information criterion
BMI: body mass index
CIs: confidence intervals
ER: estrogen receptor
FTPs: full term pregnancies
HWE: Hardy-Weinberg equilibrium
MEC: Hawai-Los Angeles Multiethnic Cohort study
HER2/neu: human epidermal growth factor receptor 2
HT: menopausal hormonal therapy
ORs: odds ratios
OC: oral contraceptives
PAK-BCCC: Pakistani breast cancer case-control study
PR: progesterone receptor
RFLP: restriction fragment length polymorphism
SNP: single nucleotide polymorphism
UV: ultraviolet
VDR: vitamin D receptor

## References

[1] Rashid MU, Zaidi A, Torres D, Sultan F, Benner A, Naqvi B, et al Prevalence of BRCA1 and BRCA2 mutations in Pakistani breast and ovarian cancer patients. Int J Cancer 2006 12 15;119(12):2832–9. 16998791 10.1002/ijc.22269

[2] Rashid MU, Gull S, Asghar K, Muhammad N, Amin A, Hamann U. Prevalence of TP53 germ line mutations in young Pakistani breast cancer patients. Fam Cancer 2012 6;11(2):307–11. 10.1007/s10689-012-9509-7 22311583

[3] Rashid MU, Muhammad N, Faisal S, Amin A, Hamann U. Constitutional CHEK2 mutations are infrequent in early-onset and familial breast/ovarian cancer patients from Pakistan. BMC Cancer 2013;13(6):312–8. 23806170 10.1186/1471-2407-13-312 PMC3699428

[4] Rashid MU, Muhammad N, Faisal S, Amin A, Hamann U. Deleterious RAD51C germline mutations rarely predispose to breast and ovarian cancer in Pakistan. Breast Cancer Res Treat 2014 6;145(3):775–84. 10.1007/s10549-014-2972-0 24800917

[5] Lowe LC, Guy M, Mansi JL, Peckitt C, Bliss J, Wilson RG, et al Plasma 25-hydroxy vitamin D concentrations, vitamin D receptor genotype and breast cancer risk in a UK Caucasian population. Eur J Cancer 2005 5;41(8):1164–9. 15911240 10.1016/j.ejca.2005.01.017

[6] Crew KD, Gammon MD, Steck SE, Hershman DL, Cremers S, Dworakowski E, et al Association between plasma 25-hydroxyvitamin D and breast cancer risk. Cancer Prev Res (Phila) 2009 6;2(6):598–604. 19470790 10.1158/1940-6207.CAPR-08-0138 PMC3077714

[7] Abbas S, Linseisen J, Slanger T, Kropp S, Mutschelknauss EJ, Flesch-Janys D, et al Serum 25-hydroxyvitamin D and risk of post-menopausal breast cancer—results of a large case-control study. Carcinogenesis 2008 1;29(1):93–9. 17974532 10.1093/carcin/bgm240

[8] Nezbedova P, Brtko J. 1alpha,25-dihydroxyvitamin D3 inducible transcription factor and its role in the vitamin D action. Endocr Regul 2004 3;38(1):29–38. 15147236

[9] Colston KW. Vitamin D and breast cancer risk. Best Pract Res Clin Endocrinol Metab 2008 8;22(4):587–99. 10.1016/j.beem.2008.08.002 18971120

[10] Lin R, White JH. The pleiotropic actions of vitamin D. Bioessays 2004 1;26(1):21–8. 14696037 10.1002/bies.10368

[11] Jones G, Strugnell SA, DeLuca HF. Current understanding of the molecular actions of vitamin D. Physiol Rev 1998 10;78(4):1193–231. 9790574 10.1152/physrev.1998.78.4.1193

[12] Feldman D, Krishnan AV, Swami S, Giovannucci E, Feldman BJ. The role of vitamin D in reducing cancer risk and progression. Nat Rev Cancer 2014 5;14(5):342–57. 10.1038/nrc3691 24705652

[13] Maruyama R, Aoki F, Toyota M, Sasaki Y, Akashi H, Mita H, et al Comparative genome analysis identifies the vitamin D receptor gene as a direct target of p53-mediated transcriptional activation. Cancer Res 2006 5 1;66(9):4574–83. 16651407 10.1158/0008-5472.CAN-05-2562

[14] Kommagani R, Payal V, Kadakia MP. Differential regulation of vitamin D receptor (VDR) by the p53 Family: p73-dependent induction of VDR upon DNA damage. J Biol Chem 2007 10 12;282(41):29847–54. 17716971 10.1074/jbc.M703641200 PMC2771332

[15] Chen H, Reed G, Guardia J, Lakhan S, Couture O, Hays E, et al Vitamin D directly regulates Mdm2 gene expression in osteoblasts. Biochem Biophys Res Commun 2013 1 4;430(1):370–4. 10.1016/j.bbrc.2012.11.003 23149414 PMC3544976

[16] Liu M, Lee MH, Cohen M, Bommakanti M, Freedman LP. Transcriptional activation of the Cdk inhibitor p21 by vitamin D3 leads to the induced differentiation of the myelomonocytic cell line U937. Genes Dev 1996 1 15;10(2):142–53. 8566748 10.1101/gad.10.2.142

[17] Lopes N, Sousa B, Martins D, Gomes M, Vieira D, Veronese LA, et al Alterations in Vitamin D signalling and metabolic pathways in breast cancer progression: a study of VDR, CYP27B1 and CYP24A1 expression in benign and malignant breast lesions. BMC Cancer 2010;10:483 10.1186/1471-2407-10-483 20831823 PMC2945944

[18] Morrison NA, Yeoman R, Kelly PJ, Eisman JA. Contribution of trans-acting factor alleles to normal physiological variability: vitamin D receptor gene polymorphism and circulating osteocalcin. Proc Natl Acad Sci U S A 1992 8 1;89(15):6665–9. 1353882 10.1073/pnas.89.15.6665 PMC49563

[19] Gross C, Eccleshall TR, Malloy PJ, Villa ML, Marcus R, Feldman D. The presence of a polymorphism at the translation initiation site of the vitamin D receptor gene is associated with low bone mineral density in postmenopausal Mexican-American women. J Bone Miner Res 1996 12;11(12):1850–5. 8970885 10.1002/jbmr.5650111204

[20] Ingles SA, Haile RW, Henderson BE, Kolonel LN, Nakaichi G, Shi CY, et al Strength of linkage disequilibrium between two vitamin D receptor markers in five ethnic groups: implications for association studies. Cancer Epidemiol Biomarkers Prev 1997 2;6(2):93–8. 9037559

[21] Arai H, Miyamoto K, Taketani Y, Yamamoto H, Iemori Y, Morita K, et al A vitamin D receptor gene polymorphism in the translation initiation codon: effect on protein activity and relation to bone mineral density in Japanese women. J Bone Miner Res 1997 6;12(6):915–21. 9169350 10.1359/jbmr.1997.12.6.915

[22] Gnagnarella P, Pasquali E, Serrano D, Raimondi S, Disalvatore D, Gandini S. Vitamin D receptor polymorphism FokI and cancer risk: a comprehensive meta-analysis. Carcinogenesis 2014 9;35(9):1913–9. 10.1093/carcin/bgu150 25053622

[23] Raimondi S, Pasquali E, Gnagnarella P, Serrano D, Disalvatore D, Johansson HA, et al BsmI polymorphism of vitamin D receptor gene and cancer risk: a comprehensive meta-analysis. Mutat Res 2014 11;769:17–34. 10.1016/j.mrfmmm.2014.06.001 25771722

[24] Tang C, Chen N, Wu M, Yuan H, Du Y. Fok1 polymorphism of vitamin D receptor gene contributes to breast cancer susceptibility: a meta-analysis. Breast Cancer Res Treat 2009 9;117(2):391–9. 10.1007/s10549-008-0262-4 19145484

[25] Wang J, He Q, Shao YG, Ji M, Bao W. Associations between vitamin D receptor polymorphisms and breast cancer risk. Tumour Biol 2013 12;34(6):3823–30. 10.1007/s13277-013-0967-9 23900677

[26] Dorjgochoo T, Delahanty R, Lu W, Long J, Cai Q, Zheng Y, et al Common genetic variants in the vitamin D pathway including genome-wide associated variants are not associated with breast cancer risk among Chinese women. Cancer Epidemiol Biomarkers Prev 2011 10;20(10):2313–6. 10.1158/1055-9965.EPI-11-0704 21828234 PMC3189282

[27] Shahbazi S, Alavi S, Majidzadeh A, Ghaffarpour M, Soleimani A, Mahdian R. BsmI but not FokI polymorphism of VDR gene is contributed in breast cancer. Med Oncol 2013 3;30(1):393 10.1007/s12032-012-0393-7 23277283

[28] McKay JD, McCullough ML, Ziegler RG, Kraft P, Saltzman BS, Riboli E, et al Vitamin D receptor polymorphisms and breast cancer risk: results from the National Cancer Institute Breast and Prostate Cancer Cohort Consortium. Cancer Epidemiol Biomarkers Prev 2009 1;18(1):297–305. 10.1158/1055-9965.EPI-08-0539 19124512

[29] Huang Y, Lu H, Bai Y, Chen Y, Zeng Y, Pan Y. Relationship between genotypes and haplotypes of vitamin D receptor gene and breast cancer risk. J Med Res 2012; 41: 89–92.

[30] Curran JE, Vaughan T, Lea RA, Weinstein SR, Morrison NA, Griffiths LR. Association of A vitamin D receptor polymorphism with sporadic breast cancer development. Int J Cancer 1999 12 10;83(6):723–6. 10597185 10.1002/(sici)1097-0215(19991210)83:6<723::aid-ijc4>3.0.co;2-3

[31] Trabert B, Malone KE, Daling JR, Doody DR, Bernstein L, Ursin G, et al Vitamin D receptor polymorphisms and breast cancer risk in a large population-based case-control study of Caucasian and African-American women. Breast Cancer Res 2007;9(6):R84 18067661 10.1186/bcr1833 PMC2246187

[32] McCullough ML, Stevens VL, Diver WR, Feigelson HS, Rodriguez C, Bostick RM, et al Vitamin D pathway gene polymorphisms, diet, and risk of postmenopausal breast cancer: a nested case-control study. Breast Cancer Res 2007;9(1):R9 17244366 10.1186/bcr1642 PMC1851389

[33] Mishra DK, Wu Y, Sarkissyan M, Sarkissyan S, Chen Z, Shang X, et al Vitamin D receptor gene polymorphisms and prognosis of breast cancer among African-American and Hispanic women. PLoS One 2013;8(3):e57967 10.1371/journal.pone.0057967 23554871 PMC3595235

[34] Sinotte M, Rousseau F, Ayotte P, Dewailly E, Diorio C, Giguere Y, et al Vitamin D receptor polymorphisms (FokI, BsmI) and breast cancer risk: association replication in two case-control studies within French Canadian population. Endocr Relat Cancer 2008 12;15(4):975–83. 10.1677/ERC-08-0056 18719092 PMC2629179

[35] Abecasis GR, Altshuler D, Auton A, Brooks LD, Durbin RM, Gibbs RA, et al A map of human genome variation from population-scale sequencing. Nature 2010 10 28;467(7319):1061–73. 10.1038/nature09534 20981092 PMC3042601

[36] Yao S, Zirpoli G, Bovbjerg DH, Jandorf L, Hong CC, Zhao H, et al Variants in the vitamin D pathway, serum levels of vitamin D, and estrogen receptor negative breast cancer among African-American women: a case-control study. Breast Cancer Res 2012;14(2):R58 22480149 10.1186/bcr3162 PMC3446393

[37] Ingles SA, Garcia DG, Wang W, Nieters A, Henderson BE, Kolonel LN, et al Vitamin D receptor genotype and breast cancer in Latinas (United States). Cancer Causes Control 2000 1;11(1):25–30. 10680726 10.1023/a:1008979417618

[38] Antoniou AC, Easton DF. Polygenic inheritance of breast cancer: Implications for design of association studies. Genet Epidemiol 2003 11;25(3):190–202. 14557987 10.1002/gepi.10261

[39] Fuhrman BJ, Freedman DM, Bhatti P, Doody MM, Fu YP, Chang SC, et al Sunlight, polymorphisms of vitamin D-related genes and risk of breast cancer. Anticancer Res 2013 2;33(2):543–51. 23393347 PMC3866631

[40] Guy M, Lowe LC, Bretherton-Watt D, Mansi JL, Peckitt C, Bliss J, et al Vitamin D receptor gene polymorphisms and breast cancer risk. Clin Cancer Res 2004 8 15;10(16):5472–81. 15328186 10.1158/1078-0432.CCR-04-0206

[41] Ziegler A, Van SK, Wellek S. Investigating Hardy-Weinberg equilibrium in case-control or cohort studies or meta-analysis. Breast Cancer Res Treat 2011 7;128(1):197–201. 10.1007/s10549-010-1295-z 21184275

[42] Khan AA (2009) A Temporal View of Socio-Political Changes in Punjab. An International Journal of South Asian Studies Vol. 24, No.2, Jul-Dec 2009, pp. 296–321

[43] National Institute of Population Studies (NIPS) [Pakistan] and ICF International. 2013. *Pakistan Demographic and Health Survey 2012–13*. Islamabad, Pakistan, and Calverton, Maryland, USA: NIPS and ICF International.

[44] Bhopal RS, Petherick ES, Wright J, Small N. Potential social, economic and general health benefits of consanguineous marriage: results from the Born in Bradford cohort study. Eur J Public Health 2014 10;24(5):862–9. 10.1093/eurpub/ckt166 24213584

[45] Sthanadar AA, Bittles AH, Zahid M. Civil unrest and the current profile of consanguineous marriage in Khyber Pakhtunkhwa Province, Pakistan. Journal of Biosocial Science 2014; 46(5), 698–701. 10.1017/S0021932015000164 26075505

[46] Bretherton-Watt D, Given-Wilson R, Mansi JL, Thomas V, Carter N, Colston KW. Vitamin D receptor gene polymorphisms are associated with breast cancer risk in a UK Caucasian population. Br J Cancer 2001 7 20;85(2):171–5. 11461072 10.1054/bjoc.2001.1864 PMC2364044

[47] Iqbal R, Jafri L, Haroon A, Habib KA. Illuminating the dark side—vitamin D status in different localities of Karachi. J Coll Physicians Surg Pak 2013 8;23(8):604–6. 23930885

[48] Imtiaz S, Siddiqui N, Raza SA, Loya A, Muhammad A. Vitamin D deficiency in newly diagnosed breast cancer patients. Indian J Endocrinol Metab 2012 5;16(3):409–13. 10.4103/2230-8210.95684 22629509 PMC3354850

[49] Holvik K, Meyer HE, Haug E, Brunvand L. Prevalence and predictors of vitamin D deficiency in five immigrant groups living in Oslo, Norway: the Oslo Immigrant Health Study. Eur J Clin Nutr 2005 1;59(1):57–63. 15280907 10.1038/sj.ejcn.1602033

[50] Lowe NM, Mitra SR, Foster PC, Bhojani I, McCann JF. Vitamin D status and markers of bone turnover in Caucasian and South Asian postmenopausal women living in the UK. Br J Nutr 2010 6;103(12):1706–10. 10.1017/S0007114509993850 20102676

[51] Mauss D, Jarczok MN, Hoffmann K, Thomas GN, Fischer JE. Association of vitamin d levels with type 2 diabetes in older working adults. Int J Med Sci 2015;12(5):362–8. 10.7150/ijms.10540 26005370 PMC4441060

[52] Raby BA, Lazarus R, Silverman EK, Lake S, Lange C, Wjst M, et al Association of vitamin D receptor gene polymorphisms with childhood and adult asthma. Am J Respir Crit Care Med 2004 11 15;170(10):1057–65. 15282200 10.1164/rccm.200404-447OC

[53] Pfeifer M, Begerow B, Minne HW, Nachtigall D, Hansen C. Effects of a short-term vitamin D(3) and calcium supplementation on blood pressure and parathyroid hormone levels in elderly women. J Clin Endocrinol Metab 2001 4;86(4):1633–7. 11297596 10.1210/jcem.86.4.7393

[54] Deeb KK, Trump DL, Johnson CS. Vitamin D signalling pathways in cancer: potential for anticancer therapeutics. Nat Rev Cancer 2007 9;7(9):684–700. 17721433 10.1038/nrc2196

[55] Lauridsen AL, Vestergaard P, Hermann AP, Brot C, Heickendorff L, Mosekilde L, et al Plasma concentrations of 25-hydroxy-vitamin D and 1,25-dihydroxy-vitamin D are related to the phenotype of Gc (vitamin D-binding protein): a cross-sectional study on 595 early postmenopausal women. Calcif Tissue Int 2005 7;77(1):15–22. 15868280 10.1007/s00223-004-0227-5

