# Supplementary figures and images for: Association between the BsmI Polymorphism in the Vitamin D Receptor Gene and Breast Cancer Risk: Results from a Pakistani Case-Control Study
Source: PLoS One. 2015 Oct 30;10(10):e0141562. doi: 10.1371/journal.pone.0141562 (PMC4627649; doi:10.1371/journal.pone.0141562)

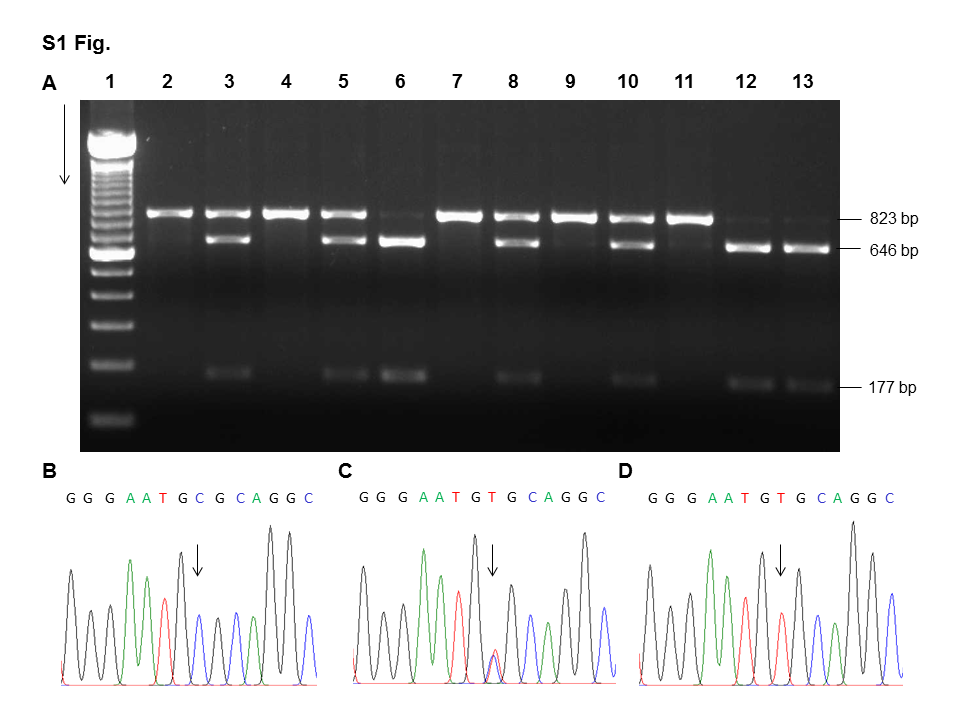

Supplement: S1 Fig — (A) PCR-RFLP products were separated on a 2% agarose gel containing ethidium bromide and scored by UV visualization. Lane 1: DNA marker (100 bp); lanes 2, 4, 7, 9, 11: AA genotype; lanes 3, 5, 8, 10: AG genotype; lanes 6, 12, 13: GG genotype (B-D) Sequencing profiles of control DNA samples showing the sequence of the reverse strand of part of intron 8 near the 3’end of the VDR gene with the C to T nucleotide change. (B) CC genotype; (C) CT genotype; (D) TT genotype. (TIF) [file pone.0141562.s001.tif]

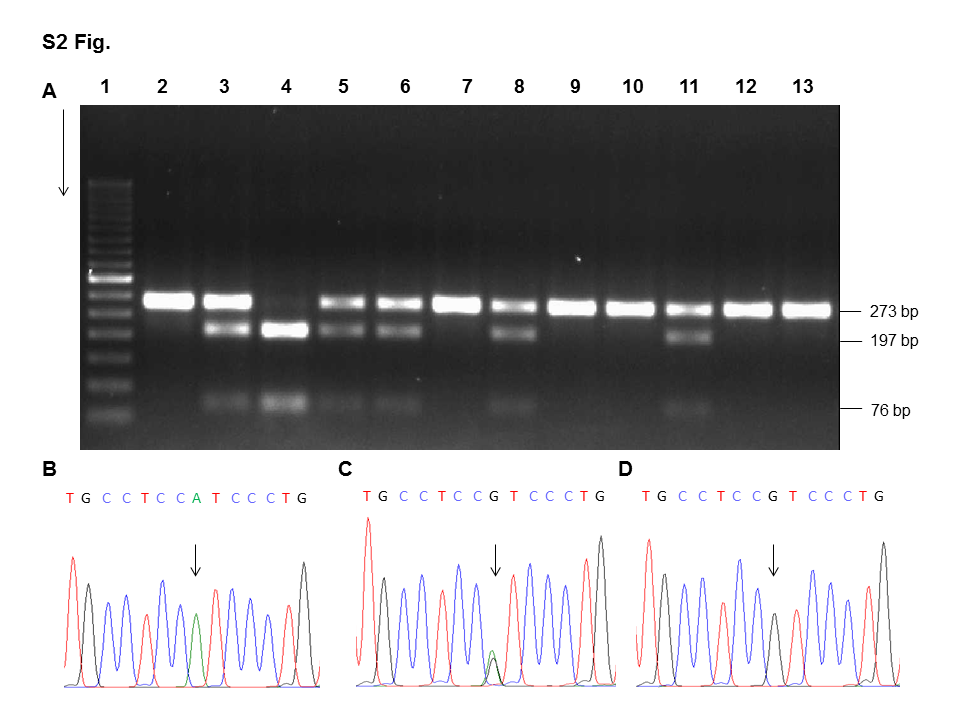

Supplement: S2 Fig — (A) PCR-RFLP products were separated on a 2% agarose gel containing ethidium bromide and scored by UV visualization. Lane 1: DNA marker (50 bp); lanes 2, 7, 9, 10, 12, 13: CC genotype; lanes 3, 5, 6, 8, 11: CT genotype; lane 4: TT genotype (B-D) Sequencing profiles of control DNA samples showing the sequence of the reverse strand of part of exon 2 of the VDR gene with the A to G nucleotide change. (B) AA genotype; (C) AG genotype; (D) GG genotype. (TIF) [file pone.0141562.s002.tif]
