# Supplementary material for: Association between the BsmI Polymorphism in the Vitamin D Receptor Gene and Breast Cancer Risk: Results from a Pakistani Case-Control Study
Source: PLoS One. 2015 Oct 30;10(10):e0141562. doi: 10.1371/journal.pone.0141562 (PMC4627649; doi:10.1371/journal.pone.0141562)
Supplement: S1 Table — (DOCX) [file pone.0141562.s003.docx]

**S1 Table.** Family history of index cases genotyped for the *VDR* *Fok*I and *Bsm*I SNPs.

| **Index cases (N=463)** | **Risk group** | **Phenotype of family^1^** |
| --- | --- | --- |
| 418 |  | Female breast cancer family |
| 220 | A1 | 1 case ≤ 30 years |
| 105 | A2 | 2 cases, >1 diagnosed ≤50 years |
| 93 | A3 | >3 cases, >1 diagnosed ≤50 years |
|  |  | Breast-ovarian cancer family |
| 45 | B | >1 breast cancer and >1 ovarian cancer |

**^1^** Including 138 families described in the initial study [1].
