# Supplementary material for: Association between the BsmI Polymorphism in the Vitamin D Receptor Gene and Breast Cancer Risk: Results from a Pakistani Case-Control Study
Source: PLoS One. 2015 Oct 30;10(10):e0141562. doi: 10.1371/journal.pone.0141562 (PMC4627649; doi:10.1371/journal.pone.0141562)
Supplement: S2 Table — (DOCX) [file pone.0141562.s004.docx]

**S2 Table.** Estimated ORs for the association of *Fok*I and *Bsm*I SNPs with breast cancer by *BRCA1/2* status (all cases *vs.* healthy controls, complete covariate model).

|  | **Healthy controls** | **Cases** | | | | | | | | |
| --- | --- | --- | --- | --- | --- | --- | --- | --- | --- | --- |
|  | **(N=752)** | **Any *BRCA1/2* status (N=463)** | | | ***BRCA1/2* non-carriers (N=353)** | | | ***BRCA1/2* carriers (N=110)** | | |
| **Genotype/Allele** | **n (%)** | **n (%)** | **OR (95% CI)**^1^ | ***P-*value**^2^ | **n (%)** | **OR (95% CI)**^1^ | ***P-*value**^2^ | **n (%)** | **OR (95% CI)**^1^ | ***P-*value**^2^ |
| *Fok*I (*F>f*) |  |  |  |  |  |  |  |  |  |  |
| *FF* | 466 (62.0) | 284 (61.3) | 0.98 (0.79-1.23) | 0.89 | 218 (61.8) | 0.96 (0.75-1.24) | 0.78 | 66 (60.0) | 1.00 (0.69-1.47) | 0.98 |
| *Ff* | 252 (33.5) | 159 (34.3) |  |  | 119 (33.7) |  |  | 40 (36.4) |  |  |
| *ff* | 34 (4.5) | 20 (4.3) |  |  | 16 (4.5) |  |  | 4 (3.6) |  |  |
| *Bsm*I (*B>b*) |  |  |  |  |  |  |  |  |  |  |
| *BB* | 233 (31.0) | 118 (25.5) | 1.25 (1.06-1.48) | **0.01** | 89 (25.2) | 1.31 (1.08-1.58) | **0.005** | 29 (26.4) | 1.15 (0.87-1.53) | 0.33 |
| *Bb* | 310 (41.2) | 189 (40.8) |  |  | 141 (39.9) |  |  | 48 (43.6) |  |  |
| *bb* | 209 (27.8) | 156 (33.7) |  |  | 123 (34.8) |  |  | 33 (30.0) |  |  |

^1^Odds ratios (ORs) with corresponding 95% confidence intervals (CIs) adjusted for age, age at menarche, number of FTPs, age at first FTP, history of breast feeding,
 duration of breast feeding, menopausal status, OC use, HT use, BMI, smoking and ethnicity.

^2^Probability value based on logistic regression and an additive penetrance model. *P*-values below 0.05 are marked in bold.
