# Supplementary material for: Association between the BsmI Polymorphism in the Vitamin D Receptor Gene and Breast Cancer Risk: Results from a Pakistani Case-Control Study
Source: PLoS One. 2015 Oct 30;10(10):e0141562. doi: 10.1371/journal.pone.0141562 (PMC4627649; doi:10.1371/journal.pone.0141562)
Supplement: S4 Table — (DOCX) [file pone.0141562.s006.docx]

**S4 Table. Estimated ORs for the association of *Fok*I and *Bsm*I SNPs with breast cancer by histopathological tumor characteristics.**

|  |  | **Morphology** | | |  | **Tumor size** | |  | **Nodal status** |  | **Histological grade** | | **ER status** |  | **PR status** |  | **HER2/neu status** |  |
| --- | --- | --- | --- | --- | --- | --- | --- | --- | --- | --- | --- | --- | --- | --- | --- | --- | --- | --- |
|  | **Cases**  **(N=463)** | **lobular vs.**  **ductal** | **ductolobular**  **vs. ductal** | **others vs. ductal** |  | **T0, T1, T2, T3, T4** | | | **≥N1 vs. N0** |  | **G1, G2, G3** |  | **positive vs. negative** | | **positive vs. negative** | | **positive vs. negative** | |
| **Genotype** | **n (%)** | **OR (95% CI)^1^** | **OR (95% CI)^1^** | **OR (95% CI)^1^** | **p-value^2^** | **OR (95% CI)^1^** | ***P*-value^2^** | | **OR (95% CI)^1^** | ***P*-value^2^** | **OR (95% CI)^1^** | ***P*-value^2^** | **OR (95% CI)^1^** | ***P*-value^2^** | **OR (95% CI)^1^** | ***P*-value^2^** | **OR (95% CI)^1^** | ***P*-value^2^** |
| *Fok*I (*F>f*) |  |  |  |  |  |  |  | |  |  |  |  |  |  |  |  |  |  |
| *FF* | 284 (61.3) | 1.43 (0.34-5.97) | 0.84 (0.22-3.22) | 1.43 (0.55-3.70) | 0.84 | 0.85 (0.57-1.27) | 0.43 | | 0.78 (0.52-1.18) | 0.24 | 0.88 (0.55-1.40) | 0.58 | 0.95 (0.64-1.41) | 0.79 | 0.95 (0.64-1.41) | 0.80 | 0.82 (0.52-1.27) | 0.37 |
| *Ff* | 159 (34.3) |  |  |  |  |  |  | |  |  |  |  |  |  |  |  |  |  |
| *ff* | 20 (4.3) |  |  |  |  |  |  | |  |  |  |  |  |  |  |  |  |  |
| *Bsm*I (*B>b*) |  |  |  |  |  |  |  | |  |  |  |  |  |  |  |  |  |  |
| *BB* | 118 (25.5) | 0.61 (0.21-1.75) | 1.33 (0.50-3.57) | 0.53 (0.24-1.15) | 0.29 | 0.85 (0.63-1.15) | 0.30 | | 1.05 (0.79-1.43) | 0.72 | 0.97 (0.73-1.28) | 0.95 | 1.06 (0.79-1.43) | 0.67 | 1.00 (0.75-1.33) | 0.99 | 0.89 (0.66-1.22) | 0.49 |
| *Bb* | 189 (40.8) |  |  |  |  |  |  | |  |  |  |  |  |  |  |  |  |  |
| *bb* | 156 (33.7) |  |  |  |  |  |  | |  |  |  |  |  |  |  |  |  |  |

^1^Odds ratios (ORs) with corresponding 95% confidence intervals (CIs) adjusted for age, age at menarche, number of FTPs, age at first FTP, history of breast feeding, duration of breast feeding, menopausal status, OC use, HT use, BMI, smoking and ethnicity.

^2^Probability value based on binary, multinomial or ordinal logistic regression and an additive penetrance model.
